# Predictors of renal outcomes and mortality in patients with renal vein thrombosis: a retrospective multicenter study

**DOI:** 10.1007/s40620-024-02166-5

**Published:** 2024-12-31

**Authors:** Osama Nady Mohamed, Sharehan Abdelrahman Ibrahim, Shereen Mohammed Mohammed Elsaghir, Marwa Ibrahim Mohamed, Basma Fathy, Ahmed M. Dardeer, Sayed Shehata, Hassan M. H. Mohammed, Amr Setouhi, Ayat Mostafa Mohamed Ahmed, Asmaa Khalf Kamel, Doaa Elzaeem Ismail, Nehal I. Abbas, Ahmed Fathy Kamel Ziady, Tamer El Zaeem Esmaeel, Ahmed S. Issa, Ahmed M. Yassin, Mostafa Mahmoud Hussein, Mostafa M. Abdelghany, Momen Mostafa Nagy, Michael Samuel Ayad, Shaimaa F. Kamel

**Affiliations:** 1 Nephrology Unit, Department of Internal Medicine, Faculty of Medicine, Minia University, Taha Hussein Street, Minia, Egypt; 2 Department of Cardiology, Faculty of Medicine, Minia University, Minia, Egypt; 3 Department of Clinical Pathology, Faculty of Medicine, Minia University, Minia, Egypt; 4 Department of Radiology, Faculty of Medicine, Minia University, Minia, Egypt; 5 Department of Vascular Surgery, Faculty of Medicine, Minia University, Minia, Egypt

**Keywords:** Renal vein thrombosis, Chronic kidney disease, Nephrotic syndrome, Malignancy

## Abstract

**Background:**

Studies on renal vein thrombosis have been conducted as case reports or case series. The renal outcomes and mortality risk of renal vein thrombosis have not been fully established. We aimed to investigate the clinical characteristics, treatment modalities, and predictors of renal outcomes and mortality in patients with renal vein thrombosis in a large multicenter cohort.

**Methods:**

We retrospectively assessed 182 patients with renal vein thrombosis diagnosed between January 2011 and May 2023 using either Doppler ultrasonography or computed tomography venography. The main outcomes analyzed were all-cause mortality, and worsening kidney function.

**Results:**

We evaluated 182 patients comprising 76 males (41.8%) and 106 females (58.2%). Nephrotic syndrome was the most common cause (51.6%) followed by malignancy (33%) and post-trauma or surgery (11%). Kidney function worsened in 126 patients (69.2%). Acute kidney injury (AKI) was identified in 72 patients (39.6%), whereas 54 patients (29.7%) developed chronic kidney disease (CKD). Multivariate logistic regression showed that declining kidney function was reliably predicted by nephrotic syndrome (Odds ratio (OR): 6.41, *P* = 0.004), serum albumin (OR: 0.31, *P* = 0.003), and diabetes mellitus (OR: 14.04, *P* < 0.001). Eighty-two patients (45.1%) died while being monitored. Sepsis accounted for the majority of deaths (25.3%). Bilateral renal vein thrombosis (Hazard Ratio (HR): 5.61, *P* < 0.001), malignancy (HR: 6.15, *P* = 0.004), serum albumin (HR: 0.12, *P* < 0.001), hemoglobin (Hb) level (HR: 0.102, *P* < 0.001) and diabetes mellitus (HR: 2.42, *P* = 0.007) were all reliable predictors of all-cause mortality using multivariate Cox regression.

**Conclusion:**

Renal vein thrombosis is associated with a higher risk of mortality and worsening kidney function. It is essential to promptly identify high risk patients and start early treatment to prevent unfavorable outcomes.

**Graphical abstract:**

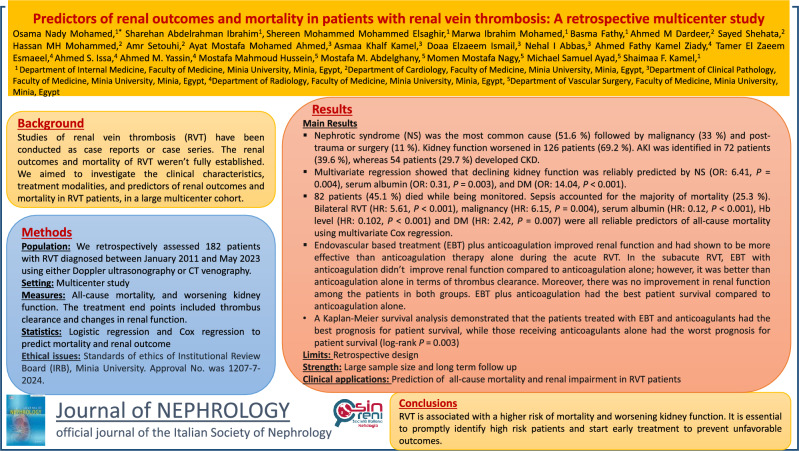

**Supplementary Information:**

The online version contains supplementary material available at 10.1007/s40620-024-02166-5.

## Introduction

Renal vein thrombosis is a rare clinical entity resulting in partial or complete obstruction of the renal veins [1]. Renal vein thrombosis is most commonly caused by hypercoagulability resulting from nephrotic syndrome, with the most prevalent disease being membranous nephropathy [2]. Hypercoagulability can be caused by a variety of factors, including disseminated malignancy, sepsis, puerperium, anti-phospholipid syndrome, Behçet’s disease, systemic lupus erythematosus, oral contraceptive pills, and intrinsic hypercoagulable states, such as antithrombin III deficiency, protein S deficiency, protein C deficiency, prothrombin G20210A mutation, and factor V Leiden mutation [2–5]. The main cause of hypercoagulability in nephrotic syndrome is increased hepatic synthesis of procoagulant factors (fibrinogen, factor V, and factor VIII) in conjunction with glomerular loss of profibrinolytic and anticoagulant proteins. Conditions characterized by decreased blood flow, elevated interstitial pressure, and endothelial damage cause an increase in plasma viscosity. Furthermore, in cases of heavy proteinuria, antithrombin III, factors XI, XII, and plasminogen are lost in the urine. Hypoalbuminemia has led to a rise in procoagulant factors, such as factors V, VII, VIII, and the von Willebrand factor, as they are often bound to albumin [6]. Renal vein invasion by tumors, most commonly renal cell carcinomas, can result in renal vein thrombosis. The inferior vena cava may be affected if the tumor is large. Even in the absence of vein invasion, extrinsic compression from mass effect can still result in a prothrombotic milieu [7]. Furthermore, endothelial damage from blunt trauma, trauma during venography, acute transplant rejection, and vasculitis can all lead to renal vein thrombosis [2, 8–10].

The prevalence of renal vein thrombosis in adults is unknown because the condition is usually asymptomatic. Furthermore, there is a high degree of variability and the reported prevalence of renal vein thrombosis in patients with nephrotic syndrome ranges from 5% to over 60% [11]. The clinical manifestations of renal vein thrombosis in adults depend on the extent, rate, and completeness of thrombosis. Patients frequently have no symptoms but patients with acute renal vein thrombosis may experience general malaise, flank pain and tenderness, hematuria, or hypertension. Acute kidney injury (AKI) may be encountered in rare severe cases (usually bilateral) [12–15]. In chronic renal vein thrombosis, patients may remain asymptomatic and present gradual deterioration of kidney function [13, 14].

A number of factors influence the prognosis of renal vein thrombosis, such as baseline kidney function, adequacy of management, severity of the underlying disease, the speed of onset of renal vein thrombosis, and bilateral involvement [2, 16–18]. Sepsis, recurrent thromboembolism, pulmonary embolism and kidney failure were causes of increased mortality. The availability of dialysis, new diagnostic techniques, and appropriate use of anticoagulants have contributed to an improvement in the prognosis of renal vein thrombosis over the past few decades [14].

Epidemiologic research on renal vein thrombosis has been carried out on case reports or case series [15, 19, 20], and population-based studies are lacking. Small sample sizes and insufficient follow-up are also limitations of these studies. We aimed to investigate the clinical characteristics, etiology, treatment protocols, and different predictors of renal outcomes and mortality in patients with renal vein thrombosis, in a large, multicenter cohort.

## Patients and methods

### Study design

This retrospective multicenter study was carried out in three tertiary University hospitals, and three central hospitals in Minia, Egypt. The hospital records were analyzed for the period between January 2011 and May 2023 to identify patients with renal vein thrombosis who were monitored for at least a year. Our hospitals have implemented clinical data repositories as a tool for researchers. The electronic health record systems are the primary source of data used in these clinical data repositories. The data elements available in these clinical data repositories include the ones that are usually recorded as discrete and coded elements in the electronic health records, such as the patient’s demographics, clinical characteristics, diagnoses, laboratory test results, medications, and diagnostic and therapeutic procedures. The study adhered to the standards of ethics of the Institutional Review Board (IRB), Faculty of Medicine, Minia University, and was assigned approval number 1207-7-2024. All study participants provided informed consent. One hundred eighty-two adult patients with renal vein thrombosis identified by computed tomography venography of renal veins or renal Doppler ultrasonography, who were above the age of 18, were eligible for enrollment in this study. Kidney transplant recipients, patients under the age of 18, and those with a disease course > 30 days were excluded. We evaluated all-cause mortality as well as renal outcomes, such as AKI and chronic kidney disease (CKD). Then, we classified the patients depending on the all-cause mortality into two groups; survivors and non-survivors.

### Clinical and laboratory characteristics

We obtained and evaluated the patients’ comprehensive medical history, taking into account the symptoms as well as findings of the physical and systemic examination. The results of laboratory investigations, including complete blood count, lipid profile, urine analysis, the spot protein to creatinine ratio or 24 h proteinuria, serum albumin, random blood sugar, C-reactive protein, kidney function tests, and liver function tests, were also examined and reviewed. The estimated glomerular filtration rate (eGFR) was determined using the formula created by the Chronic Kidney Disease Epidemiology Collaboration. Protein S, protein C, prothrombin G20210A mutation, factor V Leiden mutation, and antithrombin III were all analyzed in patients suspected of having inherited hypercoagulability. In patients with anti-phospholipid syndrome, tests for lupus anticoagulant, anti-cardiolipin antibody, and B2 anti-glycoprotein antibody were also checked and evaluated. Antinuclear antibodies, anti-double-stranded DNA antibodies, serum complement levels (C3 and C4), and anti-neutrophil cytoplasmic antibodies determined by the indirect immunofluorescence test/ELISA were further evaluated and verified in patients with systemic lupus erythematosus and vasculitis.

### Imaging investigations

Chest X-ray, electrocardiogram, and abdominal ultrasounds were retrieved and reviewed in all patients. All patients suspected of having pulmonary embolism underwent two- and three-dimensional transthoracic echocardiography and computed tomography pulmonary angiography. All patients underwent Doppler ultrasound or computed tomography venography to detect renal vein thrombosis.

### Renal Doppler ultrasonography

Patients were placed in a supine position and the kidneys were examined initially using a convex ultrasound transducer operating at 5 MHz, using a Logic E9 ultrasound machine (GE Healthcare, Chalfont St Giles, UK). Renal vein thrombosis was shown on a greyscale examination as kidney enlargement with hypoechoic cortex due to edema in the early stages or as a shrunken kidney with increased echogenicity in the later stages. Applying color Doppler ultrasonography showed that the renal artery had significant resistance with an elevated resistive index, thrombus was visible within the lumen of the renal vein, venous flow was absent and arterial diastolic flow had reversed [21].

### Computed tomography venography

Using a multi-detector computed tomography Toshiba Aquilion CXL 128 Slice CT Scanner, computed tomography venography was performed as a conventional, non-oral, post–intravenous contrast-enhanced (2 ml/Kg) computed tomography at about 120–150 s after contrast injection, which was notably later than the portal venous phase. The size of the intravenous line, injection rate, degree of hydration, and cardiac output all had an impact on the results. Similar to other cases of renal vein thrombosis, the thrombosis was visualized as a filling defect on venous phase imaging following administration of intravenous contrast. Additional computed tomography venography findings included an enlarged kidney, delayed, reduced, or absent calyceal opacification in the affected kidney, persistent cortical enhancement without parenchymal enhancement, perinephric hemorrhage, and diffuse or focal changes in attenuation because of perfusion abnormalities. The afflicted renal vein became attenuated during the chronic phase of renal vein thrombosis due to the clot retraction. The presence of collateral kidney vessels was also recorded [22].

### Renal outcomes and mortality

The time interval from enrollment to death from any cause, regardless of whether it occurred in an outpatient or inpatient setting, was utilized to determine all-cause mortality [23]. Renal outcomes included AKI, CKD or end-stage kidney disease (ESKD). AKI was diagnosed as a reduction in urine volume less than 0.5 mL/kg/h for at least 6 h; an increase in serum creatinine of 1.5 times the baseline of the previous 7 days; or an increase in serum creatinine ≥ 0.3 mg/dL within 48 h [24]. CKD was identified as a decline in eGFR less than 60 mL/min/1.73 m^2^ or damage to the kidney structure lasting longer than 3 months [23]. ESKD was described as receiving chronic dialysis or having a kidney transplant.

### Treatment protocol

A multidisciplinary team of nephrologists, cardiologists, vascular surgeons, and hematologists decided treatment for each patient based on their general condition and comorbidities [1]. Therapeutic anticoagulation was given to all patients. Parenteral heparin was used to achieve initial anticoagulation regardless of whether renal vein thrombosis was unilateral, bilateral, acute, or chronic. Patients were then switched to a vitamin K antagonist as warfarin, within three to ten days. Depending on the persistence of risk factors or on recurrence of renal vein thrombosis, anticoagulant medication was administered for a minimum of one year to lifetime. Specific therapy for underlying diseases was implemented [14]. The markers of coagulation and kidney function were strictly monitored. After an average of 5 days of anticoagulation treatment, kidney function and thrombus clearance were re-evaluated by the same imaging modalities that were used for the initial diagnosis.

Endovascular treatment, including catheter-directed thrombolysis and mechanical thrombectomy, was considered in cases of treatment failure on adequate anticoagulation, bilateral renal vein thrombosis, acute decline in kidney function or extension to the inferior vena cava. Endovascular treatment was carried out under local anesthesia and renal computed tomography venography was performed using a right internal jugular vein approach. The treatment approach was based on the thrombus location. The AngioJet System (Boston Scientific, Marlborough, MA) was utilized for patients with impaired coagulation who were not suitable candidates for long-term thrombolytic therapy for percutaneous mechanical thrombectomy. Renal artery angiography was carried out using a common femoral artery access when the thrombus was found surrounding the branches of the renal veins. The combination modality (percutaneous mechanical thrombectomy followed by catheter-directed thrombolysis through the renal vein and indirect catheter thrombolysis through the renal artery) was performed when the thrombus was in the main trunk and branches of the renal veins (mixed type). The contrast agent utilized was iodixanol (32 g/50 mL), with a mean dosage of 15 g per procedure. After the thrombolytic catheter was positioned, the patients received daily urokinase infusions (4400 IU/kg). Patients’ coagulation indicators were assessed three or four times a day while receiving thrombolytic therapy. The whole blood euglobulin lysis time was the first choice, but if not available, fibrinogen concentration, prothrombin time (PT) or partial thromboplastin time (PTT), were monitored. A prolonged PTT, PT or a lower fibrinogen concentration confirmed the establishment of systemic lysis [25].

Renal vein Doppler ultrasonography or computed tomography venography was performed before discharge (after about 10 days of treatment) (Supplemental Fig. 1). Patients who had either partial or complete resolution of the thrombus were discharged from the hospital with anticoagulant treatment (warfarin) to keep their international normalized ratio (INR) between 2.0 and 3.0 [1].

### Statistical analysis

The results were analyzed using SPSS version 25. Numbers and percentages were employed with categorical data, while medians with interquartile ranges (IQR) or means with standard deviations (SD) were utilized for continuous data. Non-parametric continuous variables for two-group comparisons were assessed using the Mann–Whitney test. Categorical data were analyzed utilizing the chi-square test. All-cause mortality and declining kidney function were the dependent outcome variables, and were adjusted for independent variables including age, body mass index (BMI), diabetes mellitus, hypertension, nephrotic syndrome, sepsis, malignancy, chronic renal vein thrombosis, bilateral renal vein thrombosis, serum albumin, and hemoglobin (Hb) level. Univariate logistic regression analyses were performed to obtain the odds ratio (OR) and 95% confidence interval (CI) for worsening kidney function adjusted for the potential risk factors including age, BMI, diabetes mellitus, hypertension, nephrotic syndrome, malignancy, sepsis, bilateral renal vein thrombosis, chronic renal vein thrombosis, and serum albumin. Similarly, the hazard ratio (HR) and 95% CI for all-cause mortality, adjusted for potential risk factors such as age, BMI, diabetes mellitus, hypertension, nephrotic syndrome, malignancy, sepsis, bilateral renal vein thrombosis, chronic renal vein thrombosis, serum albumin, Hb level, and worsening kidney function, were also calculated using Cox regression analyses. Variables that showed an association with worsening kidney function or all-cause mortality with *p* < 0.05 in univariate analysis were included in the multivariate regression. Kaplan–Meier analysis was employed to assess patient survival by treatment modality and baseline disease. Paired sample t-test was utilized to compare serum creatinine levels before and after treatment in both treatment groups.

We tried to avoid selection, information, and confounding biases. Confounding was minimized by restriction (strict exclusion criteria), age and gender matching. We reviewed electronic health records to identify the patients on regular follow up.

## Results

### Clinical characteristics, treatment and clinical outcomes of the study patients

This study evaluated 182 patients with renal vein thrombosis, comprising 76 males (41.8%) and 106 females (58.2%). Sixty-two patients (34.1%) were diabetic, while 76 (41.8%) had hypertension. Nephrotic syndrome was the most common associated disease (51.6%) followed by malignancy (33%) and post-trauma or surgery (11%). Membranous nephropathy was the most prevalent cause of nephrotic syndrome, which was reported in 76 patients (41.8%), while focal segmental glomerulosclerosis (FSGS) and membrano-proliferative glomerulonephritis (MPGN) were identified in 12 (6.6%) and 6 (3.3%) patients, respectively. Acute renal vein thrombosis was observed in 68 individuals (37.4%), whereas 114 patients (62.6%) had an asymptomatic or sub-acute presentation. During follow up, 82 patients (45.1%) died, while kidney function worsened in 126 patients (69.2%). AKI was diagnosed in 72 patients (39.6%), whereas 54 (29.7%) developed CKD. The most common cause of death was sepsis, occurring in 46 patients (25.3%). Other causes of mortality included pulmonary embolism (4.4%), hemorrhagic complications (5.5%), end-stage malignancy (6.6%) and cardiovascular diseases (3.3%) (Supplemental Table 1).

**Table 1:** Demographics, clinical characteristics, treatment and clinical outcomes of survivors and non-survivors

Variables	Survivors *n* = 100	Non-survivors *n* = 82	*P* value
Age (year)	44 (15)	54 (26.25)	** < 0.001**
Sex, *n* (%)			
Male	36 (47.4%)	40 (52.6)	
Female	64 (60.4%)	42 (39.6)	0.08
BMI (Kg/m^2^)	27.10 (4.71)	23.94 (6.26)	**0.001**
DM, *n* (%)	16 (25.8%)	46 (74.2%)	** < 0.001**
HTN, *n* (%)	40 (52.6)	36 (47.4)	0.59
Etiology, *n* (%)			
NS	62 (66%)	32 (34%)	**0.002**
Malignancy	16 (26.7%)	44 (73.3%)	** < 0.001**
Post-traumatic	18 (90%)	2 (10%)	**0.001**
Inherited hypercoagulability	4 (100%)	0 (0%)	0.07
APS	0 (0%)	4 (100%)	**0.03**
SLE	0 (0%)	2 (100%)	0.12
Clinical presentation, *n* (%)			
Acute	40 (58.8%)	28 (41.2%)	0.42
Chronic (asymptomatic)	60 (52.6%)	54 (47.7%)	
Unilateral	100 (68.5%)	46 (31.5%)	
Bilateral RVT	0 (0%)	36 (100%)	** < 0.001**
Hb level, *n* (%)			
Less than 10 g/dL	26 (33.3%)	52 (66.7%)	
More than 10 g/dL	74 (71.2%)	30 (28.8%)	** < 0.001**
Serum albumin (g/dL)	1.95 (1.40)	1.90 (0.30)	**0.004**
Treatment, *n* (%)			
Anticoagulants alone	52 (61.9%)	32 (38.1%)	
Endovascular-based treatment plus anticoagulants	48 (49.0%)	50 (51.0%)	0.08
Renal outcome (worsening kidney function), *n* (%)	56 (44.4%)	70 (55.6%)	** < 0.001**
AKI	40 (55.6%)	32 (44.4%)	0.89
CKD	16 (29.6%)	38 (70.4%)	** < 0.001**

*BMI* body mass index, *DM* diabetes mellitus, *HTN* hypertension, *RVT* renal vein thrombosis, *NS* nephrotic syndrome, *APS* anti-phospholipid syndrome, *AKI* acute kidney injury, *CKD* chronic kidney disease, *n* number, % percentage

Categorical data are expressed as number (percentage), while numerical data are expressed as median (Interquartile range). Bold represents a significant *P* value

### Clinical features, treatment modalities and clinical outcomes of survivors and non-survivors

The patients were then divided into two groups; survivors (*n* = 100) and non-survivors (*n* = 82). The survivors consisted of 36 men (47.4%) and 64 women (60.4%) and had a median age of 44 years. The non-survivors’ median age was 54 years, with 42 females (39.6%) and 40 males (52.6%). The BMI of the non-survivors was lower than that of the survivors (*P* = 0.001). Furthermore, the non-survivors had a significantly higher rate of diabetes than the survivors (*P* < 0.001). Regarding the cause of renal vein thrombosis, malignancy was found in 44 non-survivors (73.3%) and in 16 survivors (26.7%), whereas 62 survivors (66%) and 32 non-survivors (34%) had nephrotic syndrome. Two non-survivors and 18 survivors had post-traumatic renal vein thrombosis. Four survivors were found to have inherited hypercoagulability. Among non-survivors, there were only four documented cases of anti-phospholipid syndrome and two cases of systemic lupus erythematosus. There was no significant difference regarding inherited hypercoagulability, or systemic lupus erythematosus between the two groups. Thirty-six non-survivors had bilateral renal vein thrombosis, which was not found in the survivors (*P* < 0.001). The non-survivor group had considerably lower serum levels of albumin and Hb in comparison to the survivor group (*P* = 0.004, and *P* < 0.001, respectively). Considering the treatment protocols, 52 survivors and 32 non-survivors received anticoagulation alone, while endovascular-based treatment plus anticoagulation was utilized in 48 survivors and 50 non-survivors. In terms of renal outcomes, kidney function significantly decreased in the non-survivors compared to the survivors (*P* < 0.001). AKI was identified in 40 survivors and in 32 non-survivors, although CKD was documented in 16 survivors and in 38 non-survivors (Tables 1, 2 and 3).

**Table 2:** Predictors of all-cause mortality in the study patients

Variables	Univariate Cox regression				Multivariate Cox regression			
	*P *value	HR	95% CI		*P *value	HR	95% CI	
			Lower	Upper			Lower	Upper
Age	** < 0.001**	1.05	1.03	1.07	**0.045**	0.96	0.93	0.99
BMI	** < 0.001**	0.88	0.82	0.93	0.36	0.95	0.85	1.06
DM	**0.001**	2.14	1.38	3.31	**0.007**	2.42	1.28	4.59
HTN	0.92	0.98	0.63	1.515				
Bilateral RVT	** < 0.001**	4.64	2.98	7.21	** < 0.001**	5.61	2.82	11.16
Chronic RVT	0.48	1.18	0.75	1.86				
Malignancy	** < 0.001**	3.53	2.25	5.52	**0.004**	6.15	1.79	21.09
Nephrotic syndrome	** < 0.001**	0.31	0.19	0.50	** < 0.001**	0.06	0.02	0.22
Sepsis	** < 0.001**	3.58	2.30	5.56	0.20	1.42	0.83	2.42
Worsening kidney function	**0.045**	1.88	1.01	3.49	0.71	1.15	0.55	2.41
Serum Albumin	**0.014**	0.54	0.33	0.88	** < 0.001**	0.12	0.05	0.27
Hb level	** < 0.001**	0.25	0.15	0.39	** < 0.001**	0.102	0.03	0.30

*BMI* body mass index, *DM* diabetes mellitus, *HTN* hypertension, *RVT* renal vein thrombosis, *Hb* hemoglobin, *CI* confidence interval, *HR* hazard ratio

Bold represents a significant *P* value

**Table 3:** Risk factors of worsening kidney function in the study patients

Variables	Univariate logistic regression				Multivariate logistic regression			
	*P *value	OR	95% CI		*P *value	OR	95% CI	
			Lower	Upper			Lower	Upper
Age	0.15	0.98	0.96	1.01				
BMI	**0.013**	1.12	1.02	1.22	0.41	0.94	0.81	1.09
DM	** < 0.001**	11.09	3.78	32.51	** < 0.001**	14.04	3.38	58.37
HTN	0.84	0.94	0.49	1.77				
Bilateral RVT	**0.01**	4.43	1.48	13.21	0.34	0.49	0.11	2.15
Chronic RVT	**0.02**	2.25	1.12	4.53	0.41	0.68	0.26	1.73
Malignancy	0.23	1.49	0.78	2.89				
Nephrotic syndrome	** < 0.001**	6.83	3.27	14.26	**0.004**	6.41	1.81	22.71
Sepsis	0.07	2.38	0.93	6.13				
Serum Albumin	** < 0.001**	0.15	0.08	0.28	**0.003**	0.31	0.14	0.68

*RVT* renal vein thrombosis, *BMI* body mass index, *DM* diabetes mellitus, *HTN* hypertension, *CI* confidence interval, *OR* Odds ratio

Bold denotes a significant *P* value

## Effect of treatment modalities on thrombus clearance and kidney function

Patients with acute renal vein thrombosis receiving endovascular-based treatment plus anticoagulation showed considerably lower serum creatinine levels after treatment than at presentation (*P* < 0.001). Furthermore, serum creatinine levels in patients receiving anticoagulation alone improved after treatment (*P* = 0.03). Endovascular-based treatment plus anticoagulation proved to be more effective than anticoagulation alone in improving kidney function (*P* = 0.047). The percentage of patients with partial or complete thrombus clearance in the endovascular-based treatment plus anticoagulation group was higher than that in the group receiving anticoagulation alone (*P* = 0.004). Among patients with chronic renal vein thrombosis, serum creatinine levels after treatment did not differ significantly from levels before treatment in either the group undergoing endovascular-based treatment plus anticoagulation (*P* = 0.1) or in the group receiving anticoagulantion alone (*P* = 0.38). The percentage of patients with complete or partial thrombus clearance was higher in the group treated with an endovascular-based treatment plus anticoagulation than in the group treated with anticoagulation alone (*P* < 0.001). No significant difference was observed in serum creatinine levels after treatment between the two treatment groups (Table 4). Kaplan–Meier analysis showed that the patients treated with endovascular-based treatment and anticoagulants had the best survival, while those receiving anticoagulants alone had the worst survival (log-rank *P* = 0.003) (Supplemental Fig. 2).

**Table 4:** Comparison of thrombus clearance and kidney function stratified by group and disease phase

Variables	Partial or complete clearance of thrombus	*P* value	Serum creatinine (mg/dL)		*P* value
			Before treatment	After treatment	
Acute phase of renal vein thrombosis (< 14 d)					
Anticoagulantion alone	24 (38.7%)	**0.004**	2.78 (1.83)	2.41 (1.41)^a^	**0.03**
Endovascular-based treatment plus anticoagulantion	38 (61.3%)		3.52 (1.02)	1.58 (1.15)^a^	** < 0.001**
Chronic phase of renal vein thrombosis (> 14 d)					
Anticoagulantion alone	44 (42.3%)	** < 0.001**	3.00 (2.68)	2.92 (2.72)	0.38
Endovascular-based treatment plus anticoagulantion	60 (57.7%)		2.63 (0.79)	2.29 (1.33)	0.1

Categorical data are expressed as number (%), while numerical data are expressed as mean (standard deviation). a: indicates a significant difference in serum creatinine after endovascular-based treatment plus anticoagulant compared to anticoagulant alone (*P* = 0.047)

Bold denotes a significant *P* value

### Treatment effect of native diseases on the renal prognosis and mortality

Since the majority of patients had either malignancy or nephrotic syndrome, we evaluated the impact of both conditions on all-cause mortality and renal prognosis. Fifty patients had renal cell carcinoma, and 10 patients had extra-renal malignancies. Diffuse lymphoma, retroperitoneal malignancy and metastatic lung cancer were found in 5, 3, and 2 patients, respectively. Regarding all-cause mortality and worsening kidney function, there was no significant difference between renal cell carcinoma and extra-renal malignancies. Twenty-nine patients with renal cell carcinoma underwent radical nephrectomy, while 21 patients received molecular targeted therapy. Five patients with diffuse lymphoma received radiotherapy and chemotherapy, while patients with retroperitoneal malignancy and metastatic lung cancer underwent palliative symptomatic treatment. Compared to patients receiving molecular targeted therapy, patients undergoing radical nephrectomy experienced a significant decline in kidney function and a higher mortality rate (*P* < 0.001 for both). Patients with extra-renal malignancies had poor renal and patient survival. Nine of the patients experienced declining kidney function and died. We found no statistically significant difference between the treatment regimens of nephrotic syndrome in terms of deteriorating kidney function and mortality (Supplemental Table 2). Kaplan–Meier analysis revealed that the patients with renal cell carcinoma who underwent radical nephrectomy had the best patient survival compared to those receiving molecular therapy (log-rank *P* = 0.02) (Supplemental Fig. 3).

## Discussion

In this study we explored the clinical features, etiology, treatment protocols and predictors of mortality and worsening kidney function in a large multicenter cohort of patients with renal vein thrombosis. Nephrotic syndrome was the most frequent cause of renal vein thrombosis in our study (51.6%), followed by malignancy (33%), post-surgery and trauma (11%), inherited hypercoagulability (2.2%), and anti-phospholipid syndrome (2.2%). This is consistent with a previous study which showed that nephrotic syndome accounted for the majority of renal vein thrombosis [1] but is in contrast to a retrospective study which reported malignancy as the most common cause (60.9%), followed by post-surgery and trauma (16.1%), and nephrotic syndrme (12.6%) [26]. Furthermore, malignancy and nephrotic syndrome were the most frequent underlying causes in other studies [27].

In our cohort, 82 patients (45.1%) died during follow up. The most common cause of death was sepsis (25.3%), usually secondary to underlying disease causing the renal vein thrombosis, mainly malignancy and nephrotic syndrome. Death caused by the consequences of renal vein thrombosis was typically due to thromboembolism, resulting in kidney failure or pulmonary embolism. We found that age, diabetes mellitus, bilateral renal vein thrombosis, malignancy, sepsis, serum albumin, and low hemoglobin levels at diagnosis were predictors of all-cause mortality. This is in line with a previous study which evaluated 39 patients with renal vein thrombosis and reported 9 deaths (28%) during follow-up, due to malignancy [28]. Another retrospective study found that 65.5% of patients with renal vein thrombosis died during follow up. Age ≥ 75 years (HR: 3.44), malignancy (HR: 5.45), and serum albumin < 3.0 g/dL (HR: 2.88) were linked to mortality risk [26]. Infection (HR: 2.4) and active malignancy (HR: 2.4) were likewise associated with death risk in another study [27]. Pulmonary embolism, kidney failure, sepsis, and recurrent thromboembolism were common causes of mortality in patients with renal vein thrombosis.

Diabetes is a major risk factor for all-cause and cardiovascular mortality, and kidney disease [29]. Anemia is likewise linked to a higher mortality risk [30, 31]. Hypoalbuminemia is likewise independently linked to mortality [32, 33].

We reported worsening kidney functions in 126 patients (69.2%). AKI was diagnosed in 72 patients (39.6%), whereas 54 patients (29.7%) developed CKD. This is in keeping with a retrospective study showing that 18 patients (21.4%) with renal vein thrombosis experienced worsening kidney function [26] and with a case series involving 27 renal vein thrombosis patients, 15 of whom experienced renal impairment (8 acute; 7 moderate) [2]. Kidney function, especially in CKD patients, was considerably worse in the non-survivors compared to the survivors. All-cause mortality was associated with worsening kidney function. Kidney function was significantly associated with diabetes mellitus, decreased serum albumin and nephrotic syndrome, in keeping with a large body of studies [34–40].

 Previous retrospective studies also showed that renal vein thrombosis patients with nephrotic syndrome had a greater risk of worsening kidney function (HR: 18.41) [6, 26, 41, 42].

 In our series, both endovascular treatment plus anticoagulation and anticoagulation alone allowed a considerable improvement in kidney function. Patients who received endovascular-based treatment plus anticoagulation also demonstrated better thrombus clearance than those receiving anticoagulation alone.

Kaplan–Meier survival analysis in our study showed that patients undergoing endovascular-based treatment plus anticoagulation had better survival. In keeping with our data, a previous case series of 6 patients reported that percutaneous catheter-directed thrombectomy with or without thrombolysis was associated with a rapid improvement in kidney function and lower morbidity [18]. The literature is however contradictory [2]. Our findings suggest that there is a limited therapeutic window for renal vein thrombosis, and that prompt diagnosis and treatment are critical for medium- and possibly long-term recovery of kidney function. However, longer follow-up is necessary to confirm this hypothesis.

In a lack of large epidemiological studies, our study provides insights into the clinical features, etiology, treatment modalities, and predictors of renal outcomes and death in a large multicenter cohort of patients with renal vein thrombosis. The limitation of our study is its retrospective design, and the limited follow-up. Furthermore, while novel oral anticoagulants have changed the treatment of renal vein thrombosis these were not employed in our series.

## Conclusion

Nephrotic syndrome was the most frequent cause of renal vein thrombosis, followed by malignancy. Worsening kidney function was reported in 126 patients (69.2%) and was associated with decreased serum albumin, diabetes mellitus, bilateral renal vein thrombosis and nephrotic syndrome. Eighty-two patients (45.1%) died during follow-up, with sepsis accounting for the majority of deaths. Age, bilateral renal vein thrombosis, diabetes mellitus, sepsis, malignancy, serum albumin and hemoglobin levels were associated with all-cause mortality. Endovascular treatment plus anticoagulation improved kidney function and was shown to be more effective than anticoagulation therapy alone in patients with acute renal vein thrombosis. In sub-acute renal vein thrombosis, endovascular-based treatment with anticoagulation did not improve kidney function compared to anticoagulation alone; however, it allowed better thrombus clearance. Endovascular-based treatment plus anticoagulation showed better patient survival compared to anticoagulation alone.

## Supplementary Information

Below is the link to the electronic supplementary material.

Supplementary file1 (DOCX 439 KB)

Supplementary file2 (PDF 204 KB)

## Data Availability

The corresponding author can provide the datasets used and/or analyzed during the current research upon reasonable request.

## Abbreviations

AKI: Acute kidney injury
ANA: Antinuclear antibodies
ANCA: Anti-neutrophil cytoplasmic antibodies
APS: Anti-phospholipid syndrome
ARF: Acute renal failure
BMI: Body mass index
CBC: Complete blood count
CDRs: Clinical data repositories
CDT: Catheter directed thrombolysis
CI: Confidence interval
CKD: Chronic kidney disease
CTV: Computed tomographic venography
CVD: Cardiovascular disease
DM: Diabetes mellitus
EBT: Endovascular based treatment
eGFR: Estimated glomerular filtration rate
EHR: Electronic health record
ESKD: End stage kidney disease
FSGS: Focal segmental glomerulosclerosis
HR: Hazard ratio
HTN: Hypertension
ICT: Indirect catheter thrombolysis
IF: Immunofluorescence
IGR: Interquartile ranges
IRB: Institutional Review Board
IVC: Inferior vena cava
MDCT: Multi-detector computed tomography
MN: Membranous nephropathy
MPGN: Membrano-proliferative glomerulonephritis
NOACs: Novel oral anticoagulants
NS: Nephrotic syndrome
OR: Odds ratio
PMT: Percutaneous mechanical thrombectomy
PT: Prothrombin time
PTT: Partial thromboplastin time
RCC: Renal cell carcinoma
RVT: Renal vein thrombosis
SD: Standard deviation
VKA: Vitamin K antagonist
