# Supplementary material for: Predictors of renal outcomes and mortality in patients with renal vein thrombosis: a retrospective multicenter study
Source: J Nephrol. 2024 Dec 31;38(2):551–61. doi: 10.1007/s40620-024-02166-5 (PMC11961497; doi:10.1007/s40620-024-02166-5)
Supplement: Supplementary file 1 — Supplementary file1 (DOCX 439 KB) [file 40620_2024_2166_MOESM1_ESM.docx]

**
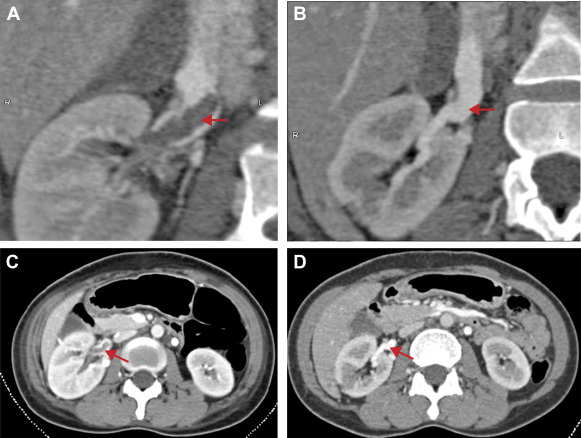
**

Supplemental Fig 1. Contrast-enhanced images of the renal veins on computed tomographic venography (CTV) demonstrating thrombosis of the right main renal vein (arrow; coronal plane [**A**] and transverse plane [**C**]). Repeat CTV after 1 week of anticoagulation plus mechanical thrombectomy revealing the right renal vein thrombus had cleared (coronal plane [**B**] and transverse plane [**D**]).


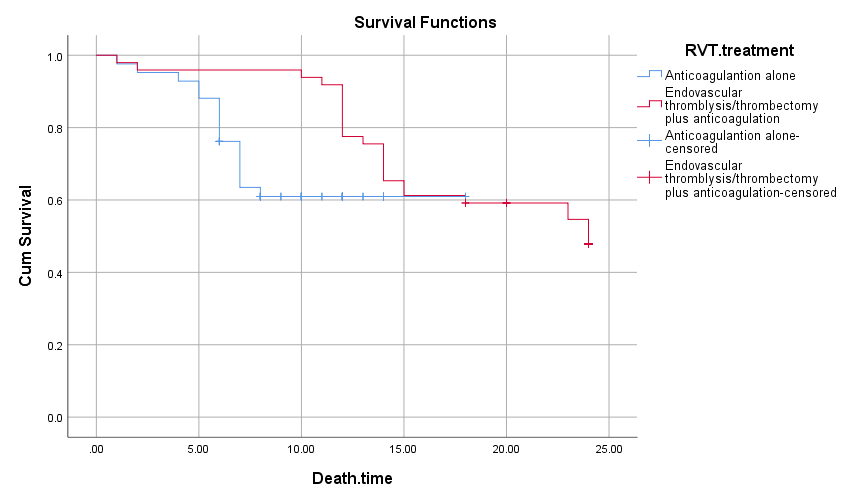


**Supplemental Fig 2.** Kaplan-Meier analysis revealing that the patients receiving anticoagulation alone experienced a notably greater all-cause mortality rate compared to those receiving EBT with anticoagulation (log-rank *P* = 0.003).


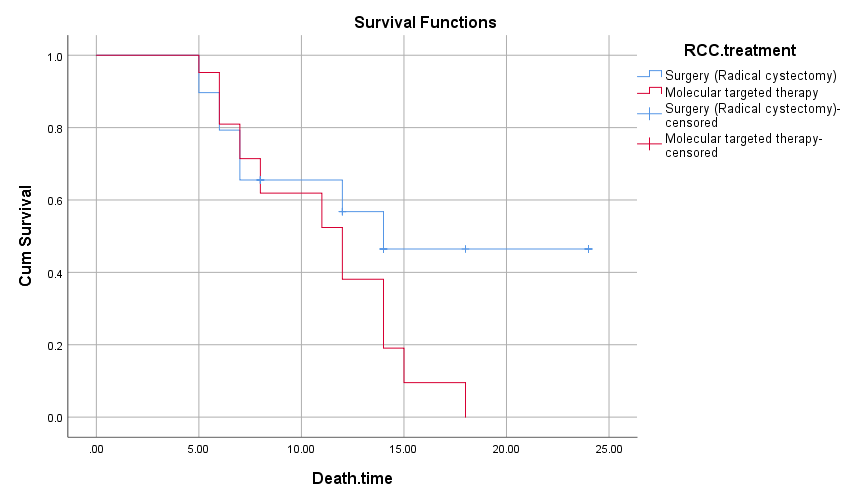


**Supplemental Fig 3**. Kaplan Meier survival analysis showing that the patients with RCC who underwent radical cystectomy had the best patient survival compared to those receiving molecular targeted therapy (log-rank *P* = 0.02).

Supplemental Table 1. Demographics, clinical characteristics, treatment modalities and clinical outcomes of the studied patients with RVT

| **Variables** | **RVT patients**  **n= 182** |
| --- | --- |
| Age (yr) | 47.07 (12.39) |
| Sex, *n* (%)  Male  Female | 76 (41.8 %)  106 (58.2 %) |
| BMI (Kg/m^2^) | 25.87 (3.76) |
| DM, *n* (%) | 62 (34.1 %) |
| HTN, *n* (%) | 76 (41.8) |
| Etiology, *n* (%)   1. NS  - MN - FSGS - MPGN  1. Malignancy 2. Post-traumatic 3. Inherited hypercoagulability 4. APS 5. SLE | 94 (51.6 %)  76 (41.8 %)  12 (6.6 %)  6 (3.3 %)  60 (33 %)  20 (11 %)  4 (2.2 %)  4 (2.2 %)  2 (1.1 %) |
| Clinical presentation, *n* (%)   - Acute - Chronic (Asymptomatic) - Unilateral - Bilateral RVT | 68 (37.4 %)  114 (62.6 %)  146 (80.2 %)  36 (19.8 %) |
| Hb level, *n* (%)   - Less than 10g/dL - More than 10g/dL | 78 (42.9 %)  104 (57.1 %) |
| Serum albumin (g/dL) | 2.2 (0.74) |
| Treatment, *n* (%)   1. Anticoagulants alone 2. EBT plus anticoagulants  - Catheter directed thrombolysis - Mechanical thrombectomy | 84 (46.2 %)  98 (53.8%)  34 (18.7 %)  64 (35.2 %) |
| Mortality, *n* (%)   - Pulmonary embolism - Sepsis - Hemorrhagic complications - End-stage malignancy - Cardiovascular cause   Renal outcome (Worsening kidney function), *n* (%)   1. AKI 2. CKD | 82 (45.1 %)  8 (4.4 %)  46 (25.3 %)  10 (5.5 %)  12 (6.6 %)  6 (3.3 %)  126 (69.2 %)  72 (39.6 %)  54 (29.7 %) |

BMI: Body mass index, DM: Diabetes mellitus, HTN: Hypertension, RVT: Renal vein thrombosis, NS: Nephrotic syndrome, APS: Anti-phospholipid syndrome, AKI: Acute kidney injury, CKD: Chronic kidney disease, EBT: Endovascular based treatment, MN: membranous nephropathy, FSGS: Focal segmental glomerulosclerosis, MPGN: Membranoproliferative glomerulonephritis. Hb: hemoglobin, Categorical data were expressed as number (percentage), while numerical data were expressed as mean (Standard deviation).

Supplemental Table 2. Effect of treatment of native disease on the renal outcome and mortality

| **Variables** | **Worsening kidney function** | | | | **Mortality** | | |
| --- | --- | --- | --- | --- | --- | --- | --- |
|  | **Yes** | | **No** | ***P* value** | **Non survivors** | **Survivors** | ***P* value** |
| Malignancy, *n*: 60 | | | | | | | |
| Malignancy type   1. Renal malignancy, *n*: 50  - Renal cell carcinoma (RCC)  1. Extra-renal malignancy  *n*: 10  - Lymphoma - Retroperitoneal malignancy - Metastatic lung cancer   Treatment modalities   1. Renal malignancy  - Surgery (Radical cystectomy) - Molecular targeted therapy  1. Extra-renal malignancy  - Radiotherapy/chemotherapy - Palliative therapy | 29 (76.3%)  9 (23.7%)  4.0  3.0  2.0  9 (31%)  20 (69%)  4 (44.4%)  5 (55.6%) | | 21 (95.5%)  1.0 (4.5%)  1.0  0.0  0.0  20 (95.2%)  1 (4.8%)  1 (100%)  0 (0.0%) | 0.06  **< 0.001**  0.29 | 35 (70%)  9 (90%)  14 (40%)  21 (60%)  4 (44.4%)  5 (55.6%) | 15 (30%)  1 (10%)  15 (100%)  0.0 (0.0%)  4 (44.4%)  5 (55.6%) | 0.19  **< 0.001**  0.29 |
| NS, *n*: 94 | | | | | | | |
| 1. Etiology of NS  - MN - FSGS - MPGN | 66 (80.5%)  10 (12.2%)  6 (7.3%) | 10 (83.3%)  2 (16.7%)  0.0 (0.0%) | | 0.6 | 32 (100%)   1. (0.0%)   0.0 (0.0%) | 44 (71%)  12 (19.4%)  6 (9.7%) | **0.003**  **0.002**  **0.03**  0.24 |
| 1. Treatment modalities  - Steroid - Calcineurin inhibitors - Mycophenolate mofetil - Cyclophosphamide - Rituximab | 12 (14.6%)  31 (37.8%)  12 (14.6%)  17 (20.7%)  10 (12.2%) | 1 (8.3%)  6 (50%)  2 (16.7%)  2 (16.7%)  1 (8.3%) | | 0.9 | 3 (9.4%)  9 (28.1%)  6 (18.8%)  9 (28.1%)  5 (15.6%) | 10 (16.1%)  28 (45.2%)  8 (12.9%)  10 (16.1%)  6 (9.7%) | 0.3 |

RCC: Renal cell carcinoma, NS: Nephrotic syndrome, MN: membranous nephropathy, FSGS: Focal segmental glomerulosclerosis, MPGN: Membranoproliferative glomerulonephritis. Bold represented a significant *P* value.
