# Supplementary material for: Predictors of renal outcomes and mortality in patients with renal vein thrombosis: a retrospective multicenter study
Source: J Nephrol. 2024 Dec 31;38(2):551–61. doi: 10.1007/s40620-024-02166-5 (PMC11961497; doi:10.1007/s40620-024-02166-5)
Supplement: Supplementary file 2 — Supplementary file2 (PDF 204 KB) [file 40620_2024_2166_MOESM2_ESM.pdf]

References

31. Guinn NR, Cooter ML, Villalpando C, Weiskopf RB (2018) Severe anemia associated with increased risk of death and myocardial ischemia in patients declining blood transfusion. Transfusion 58(10):2290-2296.

32. Di Castelnuovo A, Bonaccio M, Costanzo S, De Curtis A, Magnacca S, Persichillo M, et al (2014) The association between hypoalbuminemia and risk of death due to cancer and vascular disease in individuals aged 65 years and older: findings from the prospective Moli-sani cohort study. eClinicalMedicine (72).

33. Koene RJ, Prizment AE, Blaes A, Konety SH (2016) Shared risk factors in cardiovascular disease and cancer. Circulation 133(11):1104-1114.

34. Kang E, Han M, Kim H, Park SK, Lee J, Hyun YY, et al (2017) Baseline General Characteristics of the Korean Chronic Kidney Disease: Report from the KoreaN Cohort Study for Outcomes in Patients With Chronic Kidney Disease (KNOW-CKD). J Korean med sci 32(2):221-230.

35. Kumar M, Dev S, Khalid MU, Siddenthi SM, Noman M, John C, et al (2023) The bidirectional link between diabetes and kidney disease: mechanisms and management. Cureus 15 (9):e45615.

36. Amano H, Yoshimura K, Iijima R, Waki K, Matsumoto K, Ueda H, et al (2020) A slight decrease in the serum albumin level is associated with the rapid progression of kidney dysfunction, even within the normal range. Intern Med 59(21):2679-2685.

37. Lang J, Katz R, Ix JH, Gutierrez OM, Peralta CA, Parikh CR, et al (2018) Association of serum albumin levels with kidney function decline and incident chronic kidney disease in elders. Nephrol Dial Transplant 33(6):986-992.

38. Merlot AM, Kalinowski DS, Richardson DR (2014) Unraveling the mysteries of serum albumin-more than just a serum protein. Front Physiol 5:299.

39. Ogasawara Y, Namai T, Togawa T, Ishii K (2006) Formation of albumin dimers induced by exposure to peroxides in human plasma: a possible biomarker for oxidative stress. Biochem Biophys Res Commun 340(2):353-358.

40. Kuller LH, Eichner JE, Orchard TJ, Grandits GA, McCallum L, Tracy RP, et al (1991) The relation between serum albumin levels and risk of coronary heart disease in the Multiple Risk Factor

Edit with WPS Office

Intervention Trial. Am J Epidemiol 134(11):1266-1277.

41. Chen Y, Tang L, Feng Z, Cao X, Sun X, Liu M, et al (2014) Pathological predictors of renal outcomes in nephrotic idiopathic membranous nephropathy with decreased renal function. J Nephrol 27(3):307-316.

42. Lu H, Xiao L, Song M, Liu X, Wang F (2022) Acute kidney injury in patients with primary nephrotic syndrome: influencing factors and coping strategies. BMC Nephrol 23(1):90.

43. Lam KK, Lui CC (1998) Successful treatment of acute inferior vena cava and unilateral renal vein thrombosis by local infusion of recombinant tissue plasminogen activator. Am J Kidney Dis 32(6):1075-1079.

44. Carlsson TL, Lewis K (2018) Acute renal vein thrombosis: a case report of successful treatment with mechanical thromboaspiration. Ann Vasc Surg 51:329. e5-. e8.

Edit with WPS Office
